# Elucidating the need for prostate cancer risk calculators in conjunction with mpMRI in initial risk assessment before prostate biopsy at a tertiary prostate cancer center

**DOI:** 10.1186/s12894-024-01460-5

**Published:** 2024-03-26

**Authors:** Philipp Krausewitz, Thomas Büttner, Marthe von Danwitz, Richard Weiten, Alexander Cox, Niklas Klümper, Johannes Stein, Julian Luetkens, Glen Kristiansen, Manuel Ritter, Jörg Ellinger

**Affiliations:** 1 Department of Urology and Pediatric Urology, University Hospital Bonn, Bonn, Germany; 2 Institute of Experimental Oncology, University Hospital Bonn, Bonn, Germany; 3 Department of Diagnostic and Interventional Radiology, University Hospital Bonn, Bonn, Germany; 4 Institute of Pathology, University Hospital Bonn, Bonn, Germany

**Keywords:** Clinically significant prostate cancer, Prostate biopsy, mpMRI, Risk calculators

## Abstract

**Objective:**

Utilizing personalized risk assessment for clinically significant prostate cancer (csPCa) incorporating multiparametric magnetic resonance imaging (mpMRI) reduces biopsies and overdiagnosis. We validated both multi- and univariate risk models in biopsy-naïve men, with and without the inclusion of mpMRI data for csPCa detection.

**Methods:**

*N* = 565 men underwent mpMRI-targeted prostate biopsy, and the diagnostic performance of risk calculators (RCs), mpMRI alone, and clinical measures were compared using receiver operating characteristic curve (ROC) analysis and decision curve analysis (DCA). Subgroups were stratified based on mpMRI findings and quality.

**Results:**

csPCa was detected in 56.3%. PI-RADS score achieved the highest area under the curve (AUC) when comparing univariate risk models (AUC 0.82, *p* < 0.001). Multivariate RCs showed only marginal improvement in csPCa detection compared to PI-RADS score alone, with just one of four RCs showing significant superiority. In mpMRI-negative cases, the non-MRI-based RC performed best (AUC 0.80, *p* = 0.016), with the potential to spare biopsies for 23%. PSA-density and multivariate RCs demonstrated comparable performance for PI-RADS 3 constellation (AUC 0.65 vs. 0.60–0.65, *p* > 0.5; saved biopsies 16%). In men with suspicious mpMRI, both mpMRI-based RCs and the PI-RADS score predicted csPCa excellently (AUC 0.82–0.79 vs. 0.80, *p* > 0.05), highlighting superior performance compared to non-MRI-based models (all *p* < 0.002). Quality-assured imaging consistently improved csPCa risk stratification across all subgroups.

**Conclusion:**

In tertiary centers serving a high-risk population, high-quality mpMRI provides a simple yet effective way to assess the risk of csPCa. Using multivariate RCs reduces multiple biopsies, especially in mpMRI-negative and PI-RADS 3 constellation.

**Supplementary Information:**

The online version contains supplementary material available at 10.1186/s12894-024-01460-5.

## Introduction

There is a significant variation in the lethality of PCa, with clinically significant cases (csPCa, classified as (International Society of Urological Pathology (ISUP) Grade Group ≥ 2) exhibiting a distinct clinical course compared to non-significant cases (nsPCa, classified as ISUP grade group 1). The latter is associated with a substantial risk of overdiagnosis and overtreatment. Hence, risk stratification before a prostate biopsy is necessary to improve the balance between benefits and harms, as recently highlighted by the PROTECT study [1]. International guidelines recommend the utilization of personalized, risk-adaptive algorithms incorporating predictive prebiopsy variables in conjunction with prostate-specific antigen (PSA), or multiparametric magnetic resonance imaging (mpMRI) before deciding on a biopsy [2]. The mpMRI has emerged as a powerful predictive tool in the diagnosis of csPCa and the mpMRI-targeted biopsy has been shown to increase the detection of csPCa, while simultaneously reducing biopsies and overdiagnosis based on the Prostate Imaging Reporting and Data System (PI-RADS) [3–5].

Hence, mpMRI has been integrated into modern risk calculators (RCs), which has further enhanced their significance [6–9]. However, the heterogeneous quality of mpMRI acquisition and inter-reader variability among radiologists have implications for mpMRI reliability in PCa risk stratification [10]. To overcome these difficulties, a comprehensive quality assessment of images and systematic programs for the education and advancement of radiologists were deemed necessary [11]. Moreover, the importance of PSA-density (PSAD) in the context of mpMRI-based risk stratification was highlighted [12].

By assessing specific performance metrics, both physicians and patients can refine their decision-making regarding the need for biopsy testing. This enables them to make more informed decisions by considering the importance of employing mpMRI, adopting elaborated mpMRI-based multifaceted assessments, or opting for an economical approach relying solely on clinical parameters. Additionally, the influence of mpMRI quality on the accuracy of modern mpMRI-based RCs has not been explored to date. However, this aspect holds particular importance, given the recent findings of the MR-PROPER study, which indicated that risk-stratified systematic core needle biopsy can yield comparable rates of detecting csPCa in regions where high-quality mpMRI is not accessible [13].

This study aimed to evaluate and juxtapose the efficacy of traditional clinical risk assessment instruments against a non-MRI risk-adapted model, an exclusive mpMRI-centric risk appraisal, and mpMRI-derived multivariate risk models in the prognostication of csPCa before the initial biopsy in a tertiary prostate cancer center. In accordance with the MR-Proper-Study framework the study's evaluation encompassed differentiating mpMRI quality levels. The analysis includes PSA, PSAD, digital rectal examination (DRE), transrectal ultrasound (TRUS), PI-RADS score, as well as cutting-edge publicly accessible risk calculators developed by Radtke et al. [6], by the Mount Sinai Hospital (MSP) [9], and based on the European Randomized Study of Screening for Prostate Cancer (ERSPC) [7], both with and without mpMRI integration.

## Patients and methods

### Patients

Biopsy-naïve men who underwent systematic and mpMRI-guided biopsy were assembled from a prospectively collected institutional database (2019–2022 at the University Medical Center Bonn) and included in the ethically approved (158/22) retrospective audit. Indications for performing a biopsy were suspicious PSA, abnormal DRE, abnormal TRUS, and/or suspicious mpMRI findings defined as PI-RADS version 2.1. lesions ≥ 3 [14]. *N* = 747 men met these criteria. To facilitate the input of data into online risk calculators, the criteria established by the ERSPC risk calculator were adopted: PSA 0.4—50.0 ng/mL; Prostate volume measured by mpMRI or TRUS between 10-110 ml; Age between 50–75 years. As a result, 182 men were excluded. Hence, *n* = 565 men with complete clinical information regarding risk factors were analyzed.

### Methods

Systematic and mpMRI-targeted transrectal biopsies were performed by two highly proficient urologists (annual biopsy experience > 250, P.K., J.E.) in a single session. A standardized biopsy protocol was followed, which utilized a software-assisted (KOELIS Trinity®) template and included prophylactic antibiotics, rectal cleansing, and local anesthesia. Biopsy cores were evaluated histopathologically in accordance with international guidelines in a reference uropathology (G.K.) [2]. csPCa was defined as ISUP ≥ 2. On campus mpMRI examinations and analyses were compliant with the current American College of Radiology recommendations [14]. External mpMRI quality was not assured by on-campus reread or other measures. The cancer detection rate was determined per patient, and stratified based on the PI-RADS score, prostate volume, DRE, TRUS, PSAD, and publicly available RCs including ERSPC-RC 3 and MRI-ERSPC-RC 3 [7] (www.prostatecancer-riskcalculator.com), the MSP-RC (darasriskcalcs.shinyapps.io/MSP-RC/) [9], and the risk calculator published by Radtke et al. (Radtke-RC, formula derived) [6]. Moreover, subgroup analysis was performed, stratified based on the pre-biopsy mpMRI analysis, mimicking a plausible disparity in quality between high-volume center (= quality-assured mpMRI acquisition and analysis) and peripheral facilities (= uncertain mpMRI quality).

### Statistics

Data analysis was performed using IBM SPSS Statistics v26 and RStudio v2023.06.1 + 524. Descriptive statistics were calculated for categorical variables, including frequencies and proportions, while continuously coded variables were described with means and standard deviations. Differences were analyzed using chi-square or McNemar paired tests. Binary logistic regression was used in both univariate and multivariate analyses to identify significant predictors of overall PCa and csPCa. The diagnostic performance of predictive variables and individual variables was evaluated using receiver operating characteristic (ROC) curve analysis, and their comparisons were made using the areas under the ROC curves (AUC). The optimal threshold for sensitivity and specificity was determined using the Youden Index. AUC comparison was performed using DeLong’s Test [15]. Net benefits of the models and individual variables were analyzed using decision curve analysis (DCA) in R package “rmda” (cran.r-project.org/web/packages/rmda/). Statistical significance was established at *p* < 0.05.

## Results

PCa was detected in 62.7% with a prevalence of csPCa in 56.3% and nsPCa in 7.4% of all cases. Detailed patient characteristics are provided in Table 1.

**Table 1:** Descriptive statistics of clinical measures

Variable	All men (*n* = 565)
Age (years)	65.0 (60.0—70.0)
PSA (ng/ml)	7.8 (4.9 – 9.1)
PSAD (ng/ml/cm^3^)	0.17 (0.09 – 0.19)
Prostate volume (cm^3^)	53.7 (36.0–70.0)
Abnormal DRE (%)	198 (35.0)
Abnormal US (%)	170 (30.1)
PI-RADS 1 (%)	74 (13.1)
PI-RADS 2 (%)	31 (5.5)
PI-RADS 3 (%)	103 (18.2)
PI-RADS 4 (%)	220 (38.9)
PI-RADS 5 (%)	137 (24.2)
ISUP 1 (%)	41 (7.3)
ISUP 2 (%)	148 (26.2)
ISUP 3 (%)	85 (15.0)
ISUP 4 (%)	41 (7.3)
ISUP 5 (%)	45 (8.0)
No tumor (%)	205 (36.3)
PCa (%)	360 (62.7)
csPCa (%)	318 (56.3)
nsPCa (%)	42 (7.4)
mpMRI in house (%)	419 (74.2)
mpMRI external (%)	146 (25.8)

Table 1 shows the means and interquartile ranges or valid percentages of the collected patient data

*PSA* prostate-specific antigen, *PSAD* prostate-specific antigen density, *DRE* digital rectal examination, *US* transrectal ultrasound, *PI-RADS* The Prostate Imaging—Reporting and Data System Version 2 (PI-RADS™ v2.1), *ISUP* International Society of Urological Pathology, *CDR* cancer detection rate, *PCa*, prostate cancer, *csPCa* clinically significant prostate cancer defined as Gleason ≥ 3 + 4, *nsPCa* non-clinically significant cancer defined as Gleason ≤ 6

In univariate analysis, variables such as older age (*p* = 0.001), elevated PSA (*p* = 0.007), smaller prostate volume (*p* = 0,001, higher PSAD (*p* < 0.001), abnormal DRE and TRUS, and higher PI-RADS score (all *p* < 0.001) were associated with csPCa. However, on multivariable analysis, with the inclusion of all significant variables considered in the univariable analysis, only prostate volume (OR: 0.97; 95% CI: 0.95–0.99), PI-RADS score 4 (OR: 8.43; 95% CI: 4.27–16.64), and PI-RADS scores 5 (OR: 34.65; 95% CI: 13.10–91.65) were statistically significant predictors of csPCa.

### ROC analysis entire cohort

**Fig. 1:**
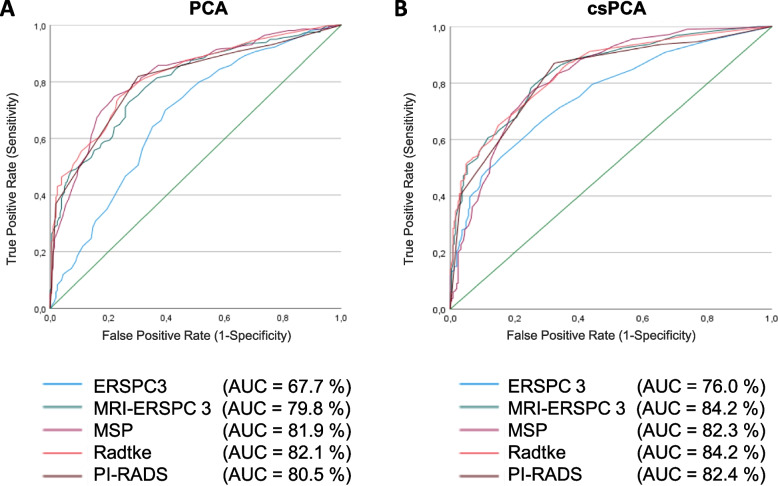
ROC curves for PCa and csPCa detection by multivariate risk models and mpMRI. ROC curve analysis of PI-RADS score, ERSPC-RC3, MRI-ERSPC-RC3, Radtke-RC and MSP-RC before initial prostate biopsy comparing healthy patients and men with proven PCa (**A**) and proven csPCa (**B**) is illustrated. In detail, ERSPC-RC3 (PCa: AUC 0.68, 95%CI 0.63–0.72, sensitivity 70%, specificity 61%; csPCa: AUC 0.76, 95%CI 0.72–0.80, sensitivity 65%, specificity 74%; MRI-ERSPC-RC3 (PCa: AUC 0.80, 95%CI 0.76–0.84, sensitivity 73%, specificity 73%; csPCa: AUC 0.84, 95%CI 0.81–0.87, sensitivity 80%, specificity 74%); MSP-RC (PCa: AUC 0.82, 95%CI 0.78–0.86, sensitivity 75%, specificity 78%; csPCa: AUC 0.82, 95%CI 0.79–0.86, sensitivity 78%, specificity 74%); Radtke-RC (PCa: AUC 0.82, 95%CI 0.78–0.86, sensitivity 75%, specificity 76%; csPCa: AUC 0.84, 95%CI 0.81–0.87, sensitivity 85%, specificity 65%); PI-RADS (PCa: AUC of 0.81,95%CI 0.77–0.84, sensitivity 82%, specificity 70%; csPCa: AUC 0.82, 95%CI 0.79–0.86, sensitivity 87%, specificity 68%)

The PSAD showed the best performance of all clinical parameters with an AUC of 0.70 (95%CI 0.65–0.74) for both PCa and csPCa. Statistically significant AUC differences were observed for PSAD compared to PSA (*p* < 0.001), DRE (*p* = 0.042), and age (*p* < 0.001). Furthermore, a significantly higher diagnostic accuracy for the PI-RADS score compared to each clinical univariate parameter for PCa (AUC 0.81 (95%CI 0.77–0.84)) and csPCa (AUC of 0.82 (95%CI 0.79–0.86)) prediction was demonstrated (all *p* < 0.001). ROC analyses of mpMRI-based risk models showed slightly improved diagnostic potential for PCa and csPCa detection. However, statistically significant AUC differences were detected only for the risk model by Radtke et al. for csPCa detection compared to the PI-RADS score (*p* = 0.019). The non-mpMRI-based ERSPC-RC3, exhibited lower diagnostic utility in detecting both PCa and csPCa (AUC 0.68 and AUC 0.76, respectively, all *p* < 0.01), Fig. 1 and Supplementary Fig. 1.

### ROC analysis stratified based on mpMRI findings

To attain a more comprehensive understanding of the predictive capacities of the mpMRI within the context of modern multivariate risk models, we conducted a ROC analysis stratified based on the PI-RADS score: PI-RADS 1–2 = negative, positive = PI-RADS 3–5, and equivocal = PI-RADS 3).

**Fig. 2:**
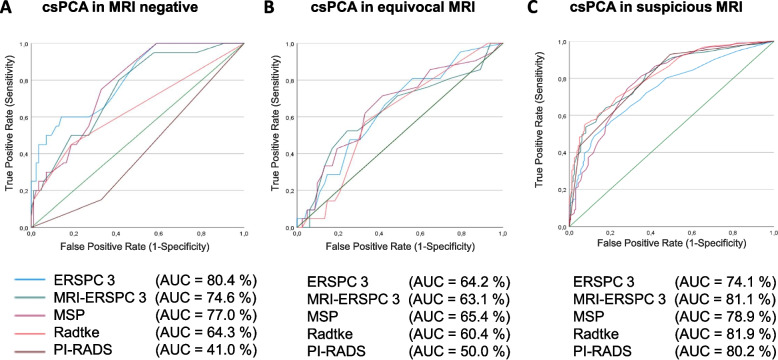
ROC curves for csPCa detection by multivariate risk models and mpMRI in stratified subgroups based on PI-RADS score. ROC curve analysis of PI-RADS score, ERSPC-RC3, MRI-ERSPC-RC3, Radtke-RC and MSP-RC before initial prostate biopsy comparing healthy patients and men with proven csPCa in men with negative mpMRI (negative multiparametric magnetic resonance tomography with PI-RADS (The Prostate Imaging—Reporting and Data System Version 2 (PI-RADS™ v2.1)) score 1–2, **A**); men with equivocal mpMRI (PI-RADS score 3, **B**); and men with suspicious mpMRI (PI-RADS score 3–5, **C**) is shown. The non mpMRI-based ERSPC-RC3 demonstrated favorable diagnostic ability for csPCa in men with negative mpMRI (AUC 0.80). It showed superior performance relative to all other risk models within this subgroup, as evidenced by a significantly higher AUC for ERSPC-RC3 in comparison to Radtke-RC (*p* = 0.016). In men PI-RADS 3 rated men the PSAD alone showed comparable performance to multivariate risk models (all *p* > 0.5: compared to Radke-RC, *p* = 0.538, ERSP-RC3, *p* = 0.850, MRI-ERSP-RC 3, *p* = 0.686 and MSP-RC, *p* = 0.934. Moreover, PSAD predicted csPCa detection better than the PI-RADS score (AUC 0.65 vs. 0.50, *p* = 0.020)

The performance of the PI-RADS score and all risk models that incorporate mpMRI into their risk assessment was decreased in individuals with negative mpMRI results and those with PI-RADS 3-rated lesions. However, the diagnostic performance of all clinical variables was also diminished in men with negative mpMRI findings and those rated as PI-RADS 3. AUCs of RCs did not differ significantly, but ERSPC-RC3 and MSP-RC showed significantly greater AUC compared to PI-RADS. Of note, ERSPC-RC3 demonstrated favorable diagnostic ability for csPCa in men with negative mpMRI (AUC 0.80). In this regard, it showed superior performance relative to all other risk models within this subgroup (Fig. 2).

Despite this, the PSAD demonstrated satisfactory performance in risk stratification for csPCa in men with negative and equivocal mpMRI (AUC 0.68 (95%CI 0.56–0.80), and AUC 0.65 (95%CI 0.52–0.77), respectively). In men harboring PI-RADS 3 rated lesions the PSAD alone showed comparable performance to multivariate risk models. Moreover, PSAD predicted csPCa detection better than the PI-RADS score (AUC 0.65 vs. 0.50, *p* = 0.020).

In contrast, in the case of positive mpMRI findings the predictive value for csPCa by both mpMRI-based RCs (AUC 0.82–0.79) and PI-RADS score alone (AUC 0.80) showed an improved predictive ability compared to the risk assessment by clinical measures. Hereby, the AUC derived from mpMRI-based risk models and the PIRADS score displayed comparable values without statistically significant distinctions. However, they all exhibited notably superior performance when contrasted with both univariate and multivariate clinical assessments with marked statistical significance (PSAD, TRUS, DRE, PSA, age all *p* < 0.001; ERSPC-RC3 *p* < 0.002, Supplementary Table 1).

### ROC analysis stratified based on mpMRI quality

**Fig. 3:**
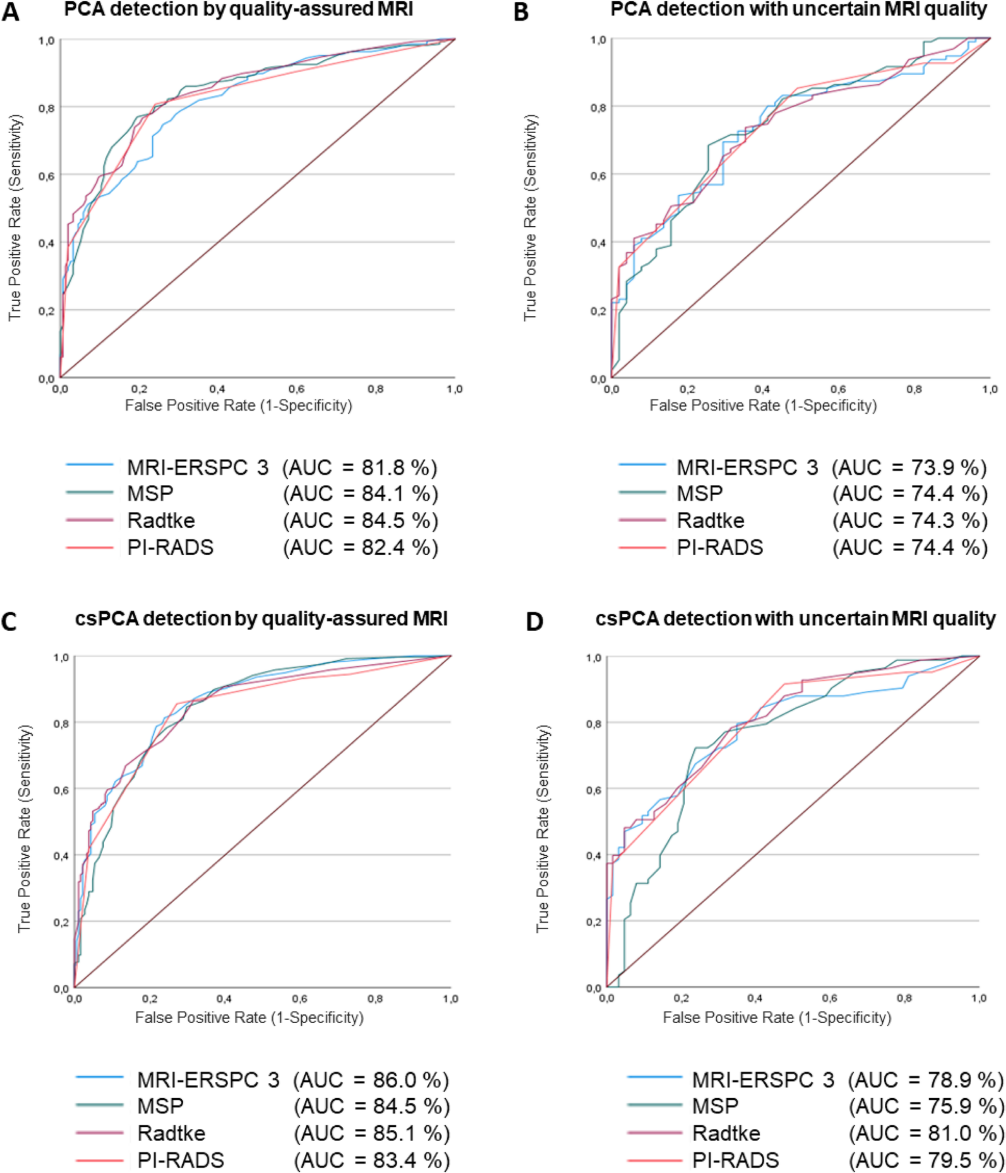
ROC curves for csPCa detection by multivariate risk models and mpMRI in stratified subgroups based on mpMRI quality. ROC curve analysis of PI-RADS score, MRI-ERSPC-RC3, Radtke-RC, and MSP-RC before initial prostate biopsy comparing healthy patients and men with proven PCa (**A**, **B**) and csPCa (**C**, **D**) in men with quality-assured mpMRI (**A**, **C**) and men with mpMRI of uncertain quality (**B**, **D**) is illustrated. Absolute mean differences in AUC for mpMRI and mpMRI-derived risk models were 0.09 and 0.08 for PCa and 0.07 and 0.04 for csPCa. However, these AUC differences remained statistically insignificant for csPCa detection with an exception for Radtke-RC and MRI-ERSPC-RC3 in PCa detection (*p* = 0.032 and *p* = 0.033, respectively)

Comparative analysis revealed that the evaluation based on a quality-assured mpMRI analysis at a high-volume center (*n* = 419) yields superior results compared to assessments made using non-quality-assured mpMRIs (*n* = 146): diagnostic accuracy by RCs (AUC 0.82–0.85) and mpMRI (AUC 0.82) on-campus vs. AUC 0.74 and 0.74 in peripheral facilities for PCa; and diagnostic ability for csPCa by RCs (AUC 0.83–0.86) and mpMRI (AUC 0.83) on-campus vs. AUC 0.76–0.81 and 0.80 in peripheral facilities, respectively (Fig. 3). Descriptive statistics of both cohorts are provided in Supplementary Table 2.

Risk stratification based solely on mpMRI demonstrated comparable results to mpMRI-based multivariate risk models, regardless image quality (Supplementary Table 3). In comparison, PCa and csPCa forecast by the non-MRI-based ERSPC-RC3 revealed a diagnostic ability of 68% (63–72%) and 76% (72–80%) in the overall cohort, respectively. Hence, using any quality mpMRI showed an improved csPCa prognostication compared to a non-MRI-based approach.

### Decision curve analysis

#### Overall cohort

No variations were detected across the models when constidering threshold probabilities spanning from 0 to 10%. Upon reaching a 20% threshold probability, a slight decrease in the need for biopsy interventions were discernible, attributable to the employment of MRI-ERSPC-RC3, MSP-RC, or independent PI-RADS score (all < 1%). At a threshold ≥ 30% csPCa risk, only 4% of biopsies could be avoided due to the use of mpMRI-based risk models (univariate and multivariate models). A detailed description is provided in Supplementary Table 4.

#### Subgroup analysis

In mpMRI-negative patients, a benefit in favor of using multivariate or univariate risk models is evident at lower risk thresholds of ≤ 10%, with a maximum potential reduction in biopsies to 3% when employing the ERSPC-RC3. The advantage of using risk models becomes more pronounced at higher risk probabilities. At a threshold of 10% csPCa risk, ERSPC-RC3 saves 23% of biopsies, MRI-ERSPC-RC 7%; Radtke-RC,MSP-RC and save none). At a threshold of 20% csPCa risk, consistent use of the models could have potentially avoided up to 39% of biopsies (ERSPC-RC3 39%; MRI-ERSPC-RC 28%; Radtke-RC 24%; and MSP-RC 18%, PSAD 21%).

In equivocal mpMRI findings, significant reductions in biopsies were observed when using the risk models starting at a 20% threshold probability: ERSPC-RC3 16%; MRI-ERSPC-RC 16%; Radtke-RC 0%; and MSP-RC 13%, PSAD 16%. Hence, the multivariate non-MRI-based risk calculator and the univariate risk model based on PSAD showed similar effectivity.

In patients with suspicious mpMRI results, there was an added value in utilizing risk models only at risk thresholds > 35%. The mpMRI-only approach consistently outperforms multivariate mpMRI-based risk models (representative benefit of additional csPCa detection at a risk threshold of 40%: Radtke-RC 1%; MRI-ERSPC-RC 6%; MSP-RC 6%; and mpMRI-only 7%).

## Discussion

The primary objective of risk stratification in the diagnosis of PCa is to minimize the necessity for biopsy examinations while upholding a robust detection rate for csPCa and a low detection rate for Gleason 6 carcinomas. This investigation scrutinized the application of different risk-based methodologies within a real-world environment, specifically within a centralized, high-capacity tertiary center focused on early detection of csPCa through mpMRI-guided biopsies, taking into account mpMRI findings and quality.

### Key findings of this inquiry encompass

PSAD emerges as the paramount univariate clinical parameter for csPCa risk classification. The PI-RADS score prevails over all clinical parameters. Multivariate non-MRI risk assessment surpasses univariate prediction models based on clinical parameters, but also falls short of isolated mpMRI evaluation and underperforms compared to mpMRI-based multivariate risk. Last, among the mpMRI-based RCs, only one surpasses an isolated csPCa risk stratification based on the PI-RADS score, irrespective of mpMRI quality. Nevertheless, assured mpMRI quality exhibits enhanced predictive capabilities for csPCa. Hence, risk stratification based on mpMRI findings alone is a simple yet efficacious course of action in a selected high-risk population.

In contrast, an added value of incorporating multivariate RCs for csPCa prediction at threshold probabilities ≥ 10%, has been demonstrated previously [6–8]. The MSP-RC was validated in the study population of the ERSPC trial with an AUC for csPCa of 0.78 [9]. In our cohort, MSP-RC demonstrated similar results (AUC 0.82); yet, the added value of using the multivariate model became only apparent at threshold levels greater than 20%. This can be attributed to our cohort, which comprises a selectively chosen patient group with substantially higher tumor burden compared to general early detection cohorts (csPCa prevalence 56% versus 38–42% in the studies by Alberts, Radtke, and Mehralivand et al. [6, 7, 16]). Henceforth, the additional expenditure of resources entailed in the execution of a multivariate risk assessment for subjects exhibiting suspicious mpMRI findings becomes a topic of uncertain relevance, especially within a previously pre-screened population. Nevertheless, the selection bias limits the generalizability of our data. Furthermore, it has to be questioned whether mpMRI-based risk calculators demonstrate improved performance when adjusted for specific csPCa prevalence. Unfortunately, the calculators under scrutiny did not provide this capability within their web interfaces.

For patients with negative mpMRI results, the optimal model for additive risk assessment is the use of the non-MRI based ERSPC-RC3. It yielded the highest reduction of biopsies. Exemplary, at a risk threshold of 10%, 23% of biopsies could be avoided. Among patients with ambiguous mpMRI findings risk stratification using the PSAD or ERSPC-RC3 stands on par with mpMRI-based multivariate models, surpassing the sole reliance on the PI-RADS score. This implies a safe use of a simplified risk model based on PSAD alone in PI-RADS 3 rated men. The value of PSAD, has previously been highlighted by others: Sigle et al. demonstrated that employing a cut off value of PSAD < 0,15 ng/ml, it is possible to avoid biopsies in up to 58% of men with PI-RADS 3 lesions [17]. Furthermore, Niu et al. developed a risk prediction model based on PSAD and the PI-RADS score showing a significantly improved diagnostic ability compared to univariate prediction [18]. Our investigation revealed comparable AUC values for PSAD and the PI-RADS score (AUC of 0.70 [95%CI 0.65–0.74] and AUC of 0.82 [95%CI 0.79–0.86]) in the overall cohort. In the context of inconclusive mpMRI results, the combination mpMRI plus PSAD outperforms individual approaches, reducing biopsies by 16% at a threshold of 20% csPCa risk. Hence, in line with the MR-PROPER trial [13], we were able to demonstrate the value of the ERSPC-RC3 and PSAD in csPCa risk stratification, particularly evident among patients with negative or ambiguous mpMRI results. In contrast to MR-Proper-Study, mpMRI-based risk stratification was markedly superior over using ERSPC-RC3 in the overall cohort and in men with suspicious mpMRI-findings. Yet, neither a significant net benefit for csPCa detection nor a significant reduction of biopsies could be achieved at clinically useful risk thresholds. Again, the current results have to be seen within the framework of evaluating a selected high-risk population at a tertiary center. On the other hand, using ERSPC-RC3 versus PI-RADS score additionally 33% of csPCa patients would have been missed. This emphasizes the mpMRI-based risk assessment in a streamlined approach for a pre-selected cohort. This is also true for patients undergoing mpMRI of uncertain quality. In the current study, additional benefits for high quality mpMRI could only be detected at threshold probabilities of > 35%. Moreover, consistent with prior research, we observed a significantly improved ability to predict significant prostate cancer in smaller prostates [19–21]. This underscores the need to consider uncertainties in both mpMRI-based and non-MRI-based risk models when dealing with larger prostate volumes.

The main limitation of this trial lies in its retrospective study design. Moreover, pre-selection bias and the absence of a gold standard correspondence with radical prostatectomy specimens need to be considered as limitations in interpreting the results. Nevertheless, it is noteworthy that in cases of increased tumor suspicions, isolated mpMRI assessment, even with uncertain quality, provides a robust prediction of csPCa in univariate risk assessment. This underscores that despite the frequently discussed limited specificity of mpMRI and its known inter-reader and inter-center variability, mpMRI currently stands as the best tool at our disposal for evaluation [22]. Nevertheless, efforts to further disseminate mpMRI, to standardize its quality and the proficiency of radiologists, are still needed. In the future, computer-aided diagnosis systems, additional imaging techniques, and modified biopsy strategies could potentially reduce variability and help to prevent biopsies, overdiagnosis and subsequently overtreatment [23–26].

## Conclusion

In tertiary centers, high-quality mpMRI serves as a clear and effective method for assessing csPCa risk. Importantly, the additional utilization of multivariate risk calculators reduces the need for multiple biopsies, especially in instances of mpMRI negativity and PI-RADS 3 constellation.

### Supplementary Information

**Supplementary Material 1.**

## Data Availability

Data is provided within the manuscript and supplementary information files.

## Abbreviations

PCa: Prostate Cancer
csPCa: Clinically significant prostate cancer
mpMRI: Multiparametric Magnetic resonance imaging
RC: Risk calculator
ROC: Receiver operating characteristic curve
DCA: Decision curve analysis
PI-RADS: Prostzate Imaging Reporting and Data System
AUC: Area under the curve
ISUP: International Society of Urological Pathology
nsPCa: Non-clinically significant prostate cancer
PSA: Prostate-specific antigen
PSAD: PSA-density
DRE: Digital rectal examination
TRUS: Transrectal ultrasound
MSP: Mount Sinai Hospital
ERSPC: European Randomized Study of Screening for Prostate Cancer
