# Supplementary material for: Elucidating the need for prostate cancer risk calculators in conjunction with mpMRI in initial risk assessment before prostate biopsy at a tertiary prostate cancer center
Source: BMC Urol. 2024 Mar 26;24:71. doi: 10.1186/s12894-024-01460-5 (PMC10964518; doi:10.1186/s12894-024-01460-5)
Supplement: Supplementary file 1 — Supplementary Material 1. [file 12894_2024_1460_MOESM1_ESM.docx]

**Elucidating the need for prostate cancer risk calculators in conjunction with mpMRI in initial risk assessment before prostate biopsy at a tertiary prostate cancer center**

P. Krausewitz^1^, T. Büttner^1^, Marthe von Danwitz^1^, R. Weiten^1^, A. Cox^1^, N. Klümper^1,2^, J. Stein^1^, J. Luetkens^3^, G. Kristiansen^4^, M. Ritter^1^, J. Ellinger^1^

^1^ Department of Urology and Pediatric Urology, University Hospital Bonn, Bonn, Germany

^2^ Institute of Experimental Oncology, University Hospital Bonn, Bonn, Germany

^3^ Department of Diagnostic and Interventional Radiology, University Hospital Bonn, Bonn, Germany

^4^ Institute of Pathology, University Hospital Bonn, Bonn, Germany

Address for correspondence: Philipp Krausewitz, M.D.

Department of Urology and Pediatric Urology,

University Hospital Bonn

Email: Philipp.krausewitz@ukbonn.de

Tel.: +4915118853551

Orcid-ID 0000-0002-8213-9975

**Supplementary Material**

In accordance with current guideline recommendations, subgroup analysis was performed for PSA ≤ 10 ng/ml. The analysis of n=459 men revealed similar results. However, all risk models exhibited increased diagnostic effectiveness when applied to this particular group. In this context, both the MRI-based models and the PI-RADS score demonstrated a markedly superior capacity for diagnostic prediction compared to the non-MRI-based model, as well as clinical parameters (all *p*<0.010) The ability to predict high-risk carcinomas (ISUP≥4) was not as robust in all univariate and multivariate risk models (**Supplementary Figure 1**).

**Supplementary Figure 1.** ROC curves for csPCa detection by multivariate risk models and mpMRI in subgroups


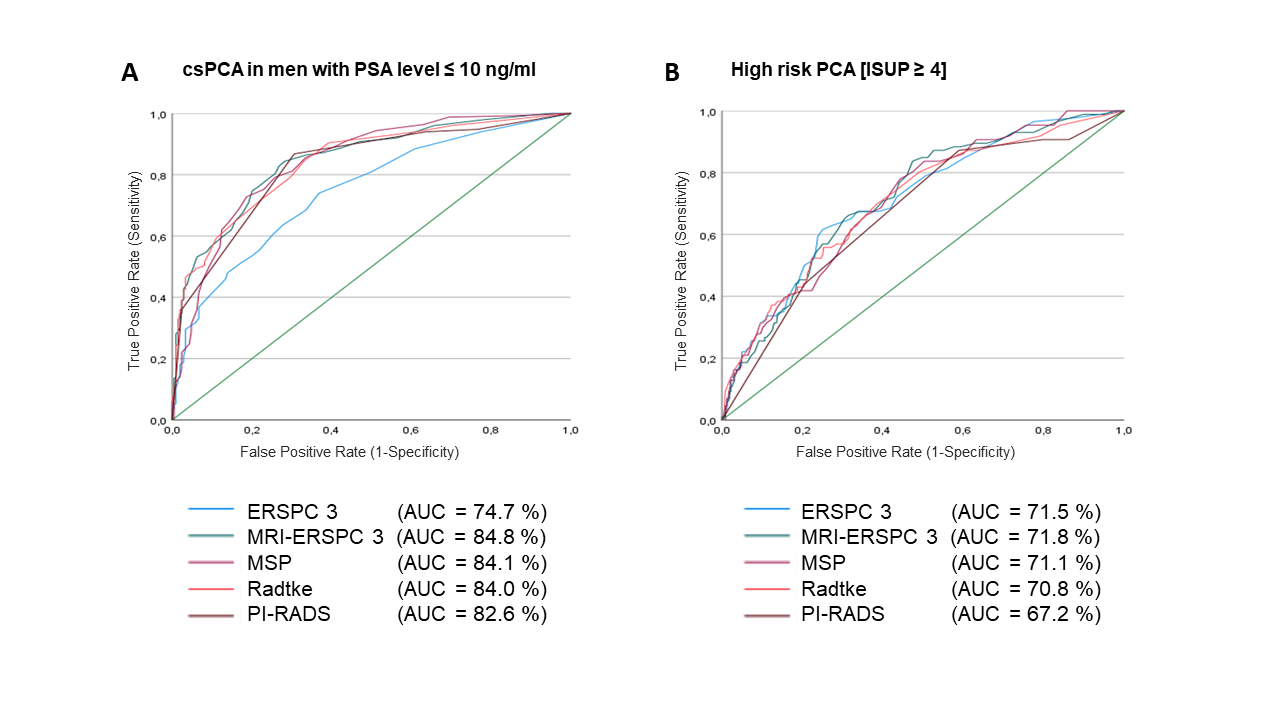


**Supplementary Figure 1.** *ROC curve analysis of PI-RADS score, ERSPC-RC3, MRI-ERSPC-RC3, Radtke-RC, and MSP-RC before initial prostate biopsy comparing healthy patients and men with proven csPCa and PSA level ≤ 10ng/ml (A) and men with proven high-risk PCa, defined as ISUP grading ≥ 4 (B).*

**Supplementary Table 1** ROC analysis of different clinical parameters for csPCa detection

| Variable | **p-value** | **AUC** | **95%CI** |
| --- | --- | --- | --- |
| All men (n=565)  Age  PSA  TRUS  DRE  Prostate volume  PSAD | 0.001*  0.007*  <0.001*  <0.001*  <0.001*  <0.001* | 0.581  0.566  0.645  0.635  0.305  0.695 | 0.534-0.628  0.518-0.613  0.600-0.690  0.589-0.681  0.261-0.349  0.652-0.738 |
| Men with negative mpMRI (n=105)  Age  PSA  TRUS  DRE  Prostate volume  PSAD | 0.001*  0.132  0.131  0.015*  0.048  0.014* | 0.734  0.609  0.609  0.675  0.358  0.677 | 0.617-0.852  0.459-0.758  0.459-0.758  0.535-0.815  0.237-0.479  0.554-0.800 |
| Men with equivocal mpMRI (n=103)  Age  PSA  TRUS  DRE  Prostate volume  PSAD | 0.851  0.707  0.719  0.778  0.119  0.034* | 0.513  0.527  0.474  0.480  0.389  0.650 | 0.360-0.667  0.387-0.666  0.339-0.610  0.343-0.617  0.255-0.524  0.525-0.776 |
| Men with suspicious mpMRI (n=460)  Age  PSA  TRUS  DRE  Prostate volume  PSAD | 0.137  0.077  <0.001*  <0.001*  <0.001*  <0.001* | 0.542  0.550  0.638  0.633  0.287  0.699 | 0.488-0.596  0.495-0.604  0.583-0.686  0.581-0.685  0.237-0.337  0.650-0.748 |

**Supplementary Table 1** shows results of the receiver operating characteristic (ROC) curve analysis including areas under the ROC curves (AUC) for csPCa detection by different clinical parameters. * represents the statistical significance of the diagnostic ability of csPCa prediction at a level p<0.05.

PSA, prostate-specific antigen; PSAD, prostate-specific antigen density; DRE, digital rectal examination; US, transrectal ultrasound; negative mpMRI, negative magnetic resonance tomography with PI-RADS (The Prostate Imaging - Reporting and Data System Version 2 (PI-RADS™ v2.1)) score 1-2; equivocal mpMRI, PI-RADS score 3; suspicious mpMRI, PI-RADS score 3-5

**Supplementary Table 2** Descriptive statistics of clinical measures of subgroups (quality assured vs. non-quality assured mpMRI)

| mpMRI quality | assured | unknown |
| --- | --- | --- |
| Participants | 419 | 146 |
| Age (years) | 65.1 ± 6.3 | 64.7 ± 6.7 |
| PSA (ng/ml) | 7.9 ± 5.3 | 7.4 ± 4.3 |
| PSAD (ng/ml/cm³) | 0.18 ± 0.17 | 0.16 ± 0.10 |
| Prostate volume (cm³) | 53.6 ± 23.3 | 54.1 ± 22.0 |
| Abnormal DRE (%) | 157 (33.3) | 41 (28.1) |
| Abnormal US (%) | 133 (30.8) | 37 (25.3) |
| PI-RADS 1 (%)  PI-RADS 2 (%)  PI-RADS 3 (%)  PI-RADS 4 (%)  PI-RADS 5 (%) | 62 (14.4)  27 (6.3)  79 (18.3)  146 (33.8)  105 (24.3) | 11 (7.5)  44 (30.1)  21 (14.4)  11 (7.5)  8 (5.5) |
| ISUP 1 (%)  ISUP 2 (%)  ISUP 3 (%)  ISUP 4 (%)  ISUP 5 (%) | 41 (7.3)  148 (26.2)  85 (15.0)  41 (7.3)  45 (8.0) | 51 (34.9)  27 (6.3)  79 (18.3)  146 (33.8)  105 (24.3) |
| No tumor (%)  PCa (%)  csPCa (%)  nsPCa (%) | 154 (35.6)  265 (61.3)  235 (54.4)  30 (7.2) | 51 (34.9)  95 (65.1)  83 (56.8)  12 (8.2) |

**Supplementary Table 2** shows means and standard deviation or valid percentages of the collected patient data for subgroup analysis dependent on the mpMRI quality.

PSA, prostate-specific antigen; PSAD, prostate-specific antigen density; DRE, digital rectal examination; the US, transrectal ultrasound; PI-RADS, The Prostate Imaging - Reporting and Data System Version 2 (PI-RADS™ v2.1); ISUP, International Society of Urological Pathology; CDR, cancer detection rate; PCa, prostate cancer; csPCa, clinically significant prostate cancer defined as Gleason ≥ 3+4; nsPCa, non-clinically significant cancer defined as Gleason ≤ 6

**Supplementary Table 3** ROC analysis of multivariate and univariate models stratified based on mpMRI-quality

| Variable | **AUC for csPCA for assured MRI quality** | **AUC unknown MRI quality** | ***P*-values**  **Assured vs. unknown quality** |
| --- | --- | --- | --- |
| All men (n=565)  ERSPC-RC3  MRI-ERSPC-RC3  MSP-RC  Radtke-RC  PI-RADS  PSAD | 0.77 (0.73-0.82)*  0.86 (0.83-0.90)*  0.85 (0.81-0.88)*  0.85 (0.82-0.89)*  0.83 (0.80-0.87)*  0.71 (0.66-0.76)* | 0.73 (0.65-0.81)*  0.79 (0.72-0.86)*  0.76 (0.68-0.84)*  0.81 (0.74-0.88)*  0.80 (0.72-0.87)*  0.65 (0.57-0.74)* | 0.362  0.078  0.062  0.302  0.298  0.281 |
| Men with negative mpMRI (n=105)  ERSPC-RC3  MRI-ERSPC-RC3  MSP-RC  Radtke-RC  PI-RADS  PSAD | 0.86 (0.76-0.95)*  0.81 (0.71-0.91)*  0.81 (0.71-0.91)*  0.60 (0.44-0.77)*  0.43 (0.28-0.58)  0.73 (0.62-0.85)* | 0.55 (0.22-0.88)  0.50 (0.15-0.85)  0.59 (0.30-0.89)  0.81 (0.53-1.00)  0.33 (0.06-0.61)  0.43 (0.05-0.81) | 0.022*  0.091  0.218  0.185  0.304  0.428 |
| Men with equivocal mpMRI (n=103)  ERSPC-RC3  MRI-ERSPC-RC3  MSP-RC  Radtke-RC  PI-RADS  PSAD | 0.65 (0.52-0.78)  0.64 (0.50-0.79)  0.67 (0.54-0.81)*  0.58 (0.45-0.72)  0.50 (0.35-0.65)  0.68 (0.56-0.81)* | 0.45 (0.14-0.77)  0.47 (0.04-0.90)  0.44 (0.11-0.78)  0.61 (0.29-0.93)  0.50 (0.14-0.86)  0.42 (0.01-0.84) | 0.571  0.630  0.509  0.865  NA  0.628 |
| Men with suspicious mpMRI (n=460)  ERSPC-RC3  MRI-ERSPC-RC3  MSP-RC  Radtke-RC  PI-RADS  PSAD | 0.74 (0.68-0.79)*  0.82 (0.77-0.87)*  0.80 (0.75-0.85)*  0.83 (0.78-0.87)*  0.81 (0.76-0.86)*  0.71 (0.65-0.77)* | 0.75 (0.71-0.86)*  0.79 (0.67-0.85)*  0.76 (0.67-0.85)*  0.80 (0.73-0.88)*  0.79 (0.72-0.87)*  0.68 (0.67-0.83)* | 0.78  0.48  0.42  0.62  0.74  0.59 |

**Supplementary Table 3** shows the results of the receiver operating characteristic (ROC) curve analysis including areas under the ROC curves (AUC) for csPCa detection by different risk models stratified by PI-RADS classification. * represents statistical significance for predicting csPCa at a level p<0.05. The third column displays the results of the comparison between the ROC results obtained with assured mpMRI quality in comparison to the ROC results based on unassured mpMRI quality

PSA, prostate-specific antigen; PSAD, prostate-specific antigen density; DRE, digital rectal examination; US, transrectal ultrasound; negative mpMRI, negative magnetic resonance tomography with PI-RADS (The Prostate Imaging - Reporting and Data System Version 2 (PI-RADS™ v2.1)) score 1-2; equivocal mpMRI, PI-RADS score 3; suspicious mpMRI, PI-RADS score 3-5

**Supplementary Table 4** Net benefit concerning csPCa detection related to using non-MRI-multivariate models, MRI-models, PI-RADS score, and PSAD at different csPCa threshold probabilities

| **Threshold** | **All** | **Radke-RC** | **ERSPC-RC3** | **MRI-ESPRC-RC3** | **MSP-RC** | **PI-RADS -Score** | **PSAD** |
| --- | --- | --- | --- | --- | --- | --- | --- |
| 0 | 0.568 |  |  |  |  |  |  |
| 5 | 0.540 |  |  |  |  |  |  |
| 10 | 0.514 |  |  |  |  | 0.495 |  |
| 15 | 0.486 |  |  |  |  | 0.473 |  |
| 20 | 0.453 |  |  | 0.461 |  | 0.456 |  |
| 25 | 0.417 |  |  | 0.437 | 0.433 | 0.432 |  |
| 30 | 0.375 |  |  | 0.420 | 0.419 | 0.405 |  |
| 35 | 0.327 | 0.411 | 0.346 | 0.406 | 0.405 | 0.373 | 0.331 |
| 40 | 0.271 | 0.378 | 0.313 | 0.379 | 0.379 | 0.336 | 0.304 |
| 45 | 0.205 | 0.313 | 0.280 | 0.354 | 0.350 | 0.374 | 0.273 |
| 50 | 0.126 | 0.276 | 0.244 | 0.317 | 0.324 | 0.349 | 0.214 |
